# Abnormal platelet function in a patient with transient eosinophilia: A case-based discussion

**DOI:** 10.51866/cr.1092

**Published:** 2026-07-07

**Authors:** Cicelia Thomas, Fatma Basyira Jamallodin, Shafini Mohamed Yusoff, Wan Suriana Wan Ab Rahman, Mohd Nazri Hassan, Zefarina Zulkafli, Salfarina Iberahim, Noor Haslina Mohd Noor, Marne Abdullah, Rosnah Bahar, Marini Ramli

**Affiliations:** 1 Department of Hematology, School of Medical Sciences, Universiti Sains Malaysia, Health Campus, Kubang Kerian, Kelantan, Malaysia.; 2 School of Dental Sciences, Universiti Sains Malaysia, Health Campus, Kubang Kerian, Kelantan, Malaysia.

**Keywords:** Eosinophilia, Platelet aggregation, Platelet function test, Ecchymosis

## Abstract

Eosinophilia is commonly associated with parasitic infections, allergic disorders and haematological conditions. Although bleeding in patients with eosinophilia is rare, platelet dysfunction may contribute, even when platelet counts are normal. We report the case of a 7-year-old boy with glucose-6-phosphate dehydrogenase deficiency who presented with recurrent spontaneous bruising over 6 months. Physical examination revealed multiple ecchymoses over the lower limbs, with no organomegaly or lymphadenopathy. Laboratory investigations revealed a normal platelet count, marked eosinophilia and prolonged bleeding time. Platelet function testing using whole blood impedance aggregometry (Chrono-Log) demonstrated reduced aggregation in response to multiple agonists, consistent with a selective impairment of aggregation to adenosine diphosphate and collagen. The patient was treated with anthelminthics, resulting in a significant reduction in the eosinophil count and resolution of bleeding symptoms. This case highlights that eosinophilia may be associated with acquired platelet dysfunction, even in the presence of normal platelet counts. Clinicians should consider platelet function testing in patients with unexplained bleeding and eosinophilia, as timely treatment of the underlying cause can lead to clinical improvement.

## Introduction

Qualitative platelet disorders are characterised by impaired platelet-dependent haemostatic function, leading to primary haemostatic dysfunction. Patients with platelet disorders typically present with mucocutaneous bleeding, such as epistaxis, gum bleeding, menorrhagia and easy bruising. Some patients may also experience excessive, early-onset haemorrhage after surgery or trauma. In contrast to quantitative thrombocytopaenia, the platelet count in these disorders is typically normal, but platelet adhesion, activation or aggregation pathways are functionally compromised. Qualitative platelet disorders can either be congenital or acquired. Among acquired cases, acquired platelet dysfunction with eosinophilia (APDE) represents a rare disorder, primarily affecting children and almost exclusively occurring in children in the South-East Asian region.^1^

APDE was historically termed ‘non-thrombocytopaenic purpura with eosinophilia’ and is distinguished by spontaneous ecchymoses with preserved platelet count and evidence of platelet dysfunction in aggregation studies.^2^ Case series of APDE have documented spontaneous bruising and reversible aggregation defects in the setting of marked eosinophilia.^3^ Another large retrospective study in children reported widespread ecchymoses with normal platelet counts and transient defects in platelet morphology and aggregation, with most cases resolving spontaneously within months.^4^ Herein, we report the case of a child presenting with recurrent spontaneous bruising and marked eosinophilia, which highlights diagnostic considerations and reinforces the importance of correlating clinical findings with targeted platelet function assessments.

## Case presentation

A 7-year-old boy presented with recurrent spontaneous bruising over the limbs for the past 6 months. The bruises predominantly affected his lower limbs bilaterally. There was no history of trauma or falls. He had undergone a tonsillectomy 2 months ago for recurrent tonsillitis. Postoperatively, bruises were noted over the jaw area, and similar bruises continued to appear over both lower limbs. The child was living with his family in a semi-detached house located in a semi-rural area. He was the second of three siblings, and none of the siblings experienced similar symptoms. The child frequently played outdoors, often in soil and occasionally barefoot. The family residence was surrounded by an unpaved compound. On physical examination, the patient was clinically well. There was no hepatosplenomegaly or lymphadenopathy. Multiple ecchymoses were present over the mandible and both lower limbs.

**Figure 1:**
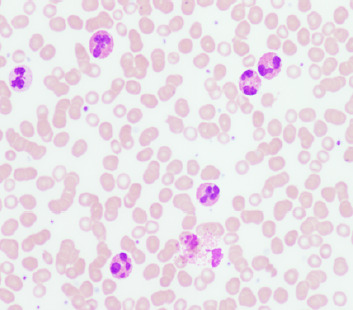
Blood film of the patient illustrating eosinophilia.

Laboratory investigations revealed a haemoglobin level of 11.4 g/dL and a total white blood cell count of 19.2x10^9^/L, with marked eosinophilia (Table 1). The platelet count was 333.0x10^9^/L. Full blood testing confirmed the findings of eosinophilia (Figure 1). The prothrombin time (PT) was within the normal range, whereas the activated partial thromboplastin time (APTT) was slightly prolonged at 47 seconds. The bleeding time measured using the conventional Ivy method was prolonged, while the factor VIII and factor IX levels were within the normal range, suggesting a defect in primary haemostasis rather than a coagulation factor deficiency. Platelet function testing was performed using whole blood impedance aggregometry (Chrono-Log aggregometer, Chrono-Log Corporation, USA). Aggregation was assessed in response to collagen (2 μg/mL and 5 μg/mL), ADP (5 μM and 10 μM), arachidonic acid (0.5 mM) and ristocetin (1 mg/mL). The results showed a selective platelet dysfunction, with impaired aggregation in response to ADP and collagen, while responses to arachidonic acid and ristocetin remained normal. The absence of parasitological confirmation represents a limitation of this case.

**Table 1:** Laboratory findings of the patient during his hospital visit.

Parameter	First visit	Second visit	Reference range
Hemoglobin level (g/dL)	11.4	11.4	11.5-15.5
Total white blood cell count (x10^9^/L)	19.20	13.32	5.00-13.00
Absolute eosinophil count (x10^9^/L)	5.95	3.22	0.10-1.00
Platelet count (x10^9^/L)	333	219	150-450
Prothrombin time (second)	14.0	14.7	12.6-15.7
Activated partial thromboplastin time (second)	47.3	46.9	30.0-45.8
Factor VIII level (%)	143	-	50-190
Factor IX level (%)	72	-	57-160
Bleeding time (minutes)	9	-	2-9

Anti-helminthic therapy was initiated empirically due to persistent eosinophilia, environmental exposure risk and residence in a helminth-endemic region. Although no stool examination was conducted to identify parasites, empirical anti-helminthic therapy was considered reasonable given the clinical context and the low risk of therapy. A marked reduction in the eosinophil count was observed on subsequent blood tests. The initial APTT was borderline prolonged but normalised on repeat testing after treatment. Clinically, there was no further spontaneous bruising.

## Discussion

This case highlights an unusual coexistence of APDE in a child, suggesting a potential mechanism that may contribute to platelet dysfunction. APDE is a rare, reversible platelet function disorder seen primarily in children in regions where parasitic infections are common such as Thailand, Malaysia and Singapore.^3^ It is characterised by intermittent mucocutaneous bleeding symptoms, most often spontaneous bruising or mucocutaneous bleeding with the presence of marked eosinophilia and normal platelet counts. Although often described as transient, symptoms may recur over several months before spontaneous resolution. The key laboratory finding is defective platelet aggregation in vitro, typically in response to agonists such as collagen and ADP, which are important activators of platelet function.^5^ The pathophysiology of APDE remains unclear but is thought to involve eosinophil-mediated modulation of platelet function; hypotheses include eosinophil cationic proteins (ECPs) inhibiting platelet aggregation and immunoglobulin-mediated effects on platelet activation. Within eosinophils, there are several granule proteins, including ECPs. ECPs cause platelet aggregation inhibition in a time-dependent manner without platelet death.^6^ Studies have shown that in cases of eosinophilia, there is a decrease in platelet aggregation induced by substances such as ADP, collagen or epinephrine.^7^

In primary care, a child presenting with recurrent bruising more commonly raises concern for conditions such as immune thrombocytopaenia, von Willebrand disease, haematological malignancy or non-accidental injury. These diagnoses appropriately warrant early exclusion. In this case, thorough clinical history-taking did not suggest features of von Willebrand disease or non-accidental injury. The presence of a normal platelet count made immune thrombocytopaenia unlikely, while the absence of circulating blasts on the peripheral blood film reduced suspicion for leukaemia. The coexistence of normal platelet counts with unexplained eosinophilia should prompt consideration of secondary platelet dysfunction, particularly in regions where parasitic exposure is common.

APDE is often associated with parasitic infections, including hookworm, *Trichuris trichiura* and *Ascaris lumbricoides*,^8,9^ and less frequently with other eosinophilic inflammatory conditions such as asthma. Prolonged bleeding time and reduced platelet aggregation could also be found in patients with eosinophilic inflammation, such as asthma, allergic rhinitis and Hay fever.^10^ In the present case, the key clinical concern for primary care was recognising that transient eosinophilia in a child with bleeding symptoms may signal a reversible secondary platelet dysfunction rather than an inherited bleeding disorder. In endemic settings, parasitic exposure should be actively considered, and careful environmental history-taking is essential. This highlights the important role of family physicians in detecting environmentally related causes of haematological abnormalities and coordinating follow- up to ensure resolution.

In the initial evaluation of bleeding in children, screening tests are essential. A normal platelet count with normal PT and APTT generally suggests intact coagulation factor pathways and shifts the diagnostic consideration towards a qualitative platelet disorder. In our case, the platelet count was normal, and the APTT was only borderline prolonged, subsequently normalising on repeat testing, making a significant coagulation factor deficiency unlikely. There is no specific haematological pattern diagnostic of APDE. However, transient eosinophilia with preserved haemoglobin level and platelet count may raise clinical suspicion when correlated with age and environmental exposure. Platelet aggregation studies are specialised laboratory investigations typically performed following referral to haematology services. They assess platelet response to agonists and help differentiate qualitative platelet dysfunction from coagulation disorders. In primary care, such testing is usually considered when unexplained mucocutaneous bleeding persists despite normal platelet count and coagulation profile. Abnormal platelet aggregation induced by collagen is the most sensitive test of platelet dysfunction in APDE, reflecting the direct effect of eosinophil-derived proteins such as ECPs on collagen-mediated platelet activation.^11^ Few case reports with abnormal platelet aggregation have been published.^3,4,12,13^ Although these aggregation findings support an acquired aetiology, similar findings of reduced aggregation to ADP, collagen and epinephrine may also occur in inherited storage pool disease (SPD).^14^

**Figure 2:**
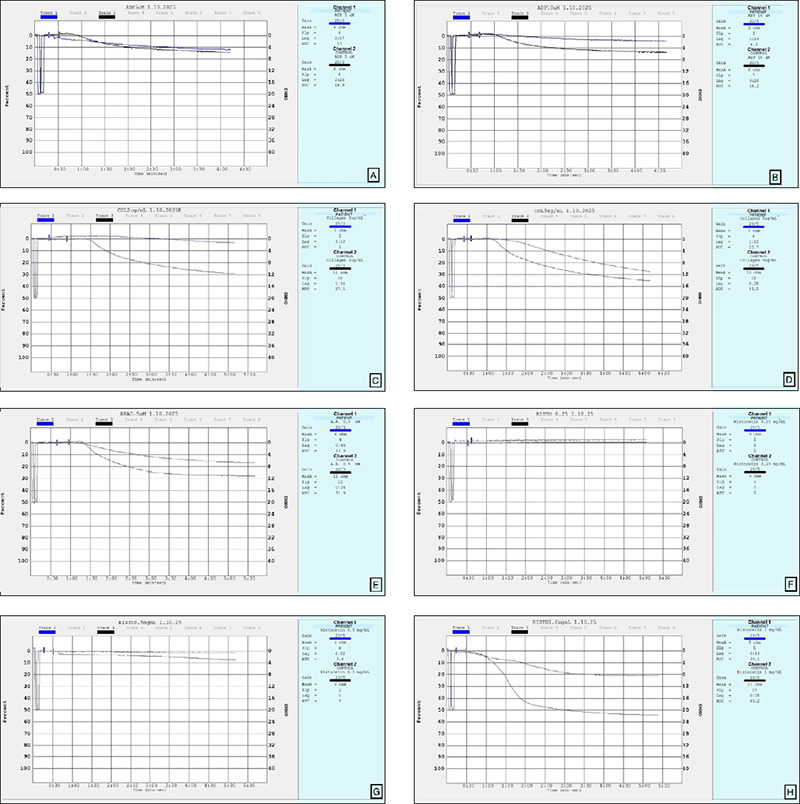
Platelet aggregation test results of the patient. (A and B) Platelet aggregation curves showing response to ADP (reduced response at a low dose of 5 uM and a high dose of 10 uM). (C and D) Platelet aggregation curves showing response to collagen (absent response at a low dose of 2 ug/mL and reduced secondary wave at a high dose of 5 ug/mL). (E) Platelet aggregation curves showing normal response to arachidonic acid. (F to H) Platelet aggregation curves showing normal response to ristocetin (markedly low dose, 0.25 mg/mL; low dose, 0.5 mg/mL; and high dose, 1.0 mg/mL). ADP: adenosine diphosphate.

In our case, the eosinophil count improved significantly following anthelminthic therapy and correlated with the resolution of bleeding symptoms, which strongly supports the diagnosis ofAPDE. However, we acknowledge the limitation that repeat platelet function testing after the normalisation of the eosinophil count was not performed, which would have provided additional confirmation of the platelet recovery and further clarified the relationship between eosinophilia and platelet dysfunction. The temporal correlation between declining eosinophilia and recovery of platelet function has been consistently documented in APDE secondary to *Trichuris* and *Ascaris* infections, with impaired aggregation response to collagen and ADP, normalising within weeks of receiving anthelminthic treatment.^15^ From a primary care perspective, empirical anti-helminthic therapy may be reasonable in children with persistent eosinophilia and environmental exposure risk in endemic settings. However, clear follow-up is essential. If eosinophilia or bleeding symptoms persist after treatment, further evaluation should include repeat full blood count testing and blood film, stool examination for parasites and consideration of referral for haematological assessment to exclude primary platelet disorders, hypereosinophilic syndromes or other systemic or malignant conditions. This case highlights the crucial role of family physicians in recognising environmentally related haematological abnormalities, initiating appropriate empirical treatment when indicated and providing structured monitoring and family guidance.

## Conclusion

APDE is a rare cause of recurrent bruising in children but should be considered when bleeding occurs alongside unexplained eosinophilia and normal platelet counts, particularly in regions where parasitic exposure is common. Empirical anti-helminthic therapy may be reasonable in such contexts, but clinicians should conduct follow-up to ensure resolution of eosinophilia and bleeding. Careful exclusion of more common or serious causes, such as immune thrombocytopaenia, haematological malignancy or non-accidental injury, remains essential. Awareness of APDE and structured monitoring can help family physicians manage these cases safely and avoid unnecessary invasive investigations.
